# A prospective cohort study of depression (PROUD) in China: rationale and design

**DOI:** 10.1007/s44194-022-00018-7

**Published:** 2023-01-09

**Authors:** Jingjing Zhou, Jinjie Xu, Rui Liu, Han Qi, Jian Yang, Tong Guo, Jia Zhou, Xuequan Zhu, Ling Zhang, Xiongying Chen, Nan Lyu, Zizhao Feng, Guofu Zhang, Min Liu, Weiwei Wang, Yun Wang, Zhifang Zhang, Le Xiao, Yuan Feng, Gang Wang

**Affiliations:** 1 Beijing Key Laboratory of Mental Disorders, National Clinical Research Center for Mental Disorders & National Center for Mental Disorders, Beijing Anding Hospital, Capital Medical University, Beijing, 100088 China; 2 Advanced Innovation Center for Human Brain Protection, Capital Medical University, Beijing, 100088 China

**Keywords:** Major depressive disorder, Cohort study, Multidimensional indicators, Multiple follow-up strategies, China

## Abstract

**Background:**

Major depressive disorder (MDD) imposes a heavy global disease burden. However, current etiology, diagnosis and treatment remain unsatisfactory and no previous study has resolved this problem. Building on the strengths and limitations of previous cohort studies of MDD, the prospective cohort study of depression (PROUD) is a 3-year large-scale cohort study designed to collect multidimensional data with a flexible follow-up schedule and strategy. The goal is to establish a nationally representative, high-quality, standardized depression cohort to support precise diagnosis and treatment of MDD and address the gap in current research.

**Methods:**

PROUD is a patient-based, nationally representative multicenter prospective cohort study with baseline and 3-year follow-up assessments. It will be carried out from January 2022 to December 2026 in 52 qualified tertiary hospitals in China. A total of 14,000 patients diagnosed with MDD, according to the DSM-5 criteria, and aged ≥ 16 years, will be recruited to PROUD. Participants aged 18-65 years who have not received any treatment during a depressive episode will be included in the precision medicine cohort (PMC) of PROUD (*n*=4,000). Patients who meet the general eligibility criteria but not the PMC criteria will be included in the naturalistic observation cohort (NOC) of PROUD (*n*=10,000). A multiple follow-up strategy, including scheduled, remote, telephone, external visits and patient self-reports, will be implemented to collect comprehensive sociodemographic, clinical information, biospecimens, neuroimaging, cognitive function and electrophysiology data and digital phenotypes according to strict standard operating procedures implemented across centers. Trial registration: ChiCTR2200059053, registered on 23 April 2022, http://www.chictr.org.cn/showproj.aspx?proj=165790.

**Conclusions:**

PROUD is a prospective cohort study of MDD patients in China. It will provide a comprehensive database facilitating further analyses and aiding the development of homeostatic and precision medicine in China.

## Introduction

Homeostasis refers to a state in which the organism maintains the relative stability of the internal environment by regulating the coordinated activities of organs and systems. Homeostasis is the basis for maintaining health and ensuring various physiological functions. Conversely, the occurrence and development of disease is usually accompanied by an imbalance in the body's homeostasis (Qin and Wang, 2022). Major depressive disorder (MDD) is a highly prevalent and debilitating mental disorder with a lifetime prevalence among Chinese adults of 6.9% (Lu *et al.*, 2021). MDD has been identified as one of the leading contributors to global disease burdens (Diseases and Collaborators, 2020). However, its etiology is not yet fully understood, and despite being the focus of more attention in recent years, diagnosis and treatment remain unsatisfactory. Firstly, there are many possible causal factors for MDD, ranging from biological to psychosocial. It is generally believed to be the result of a combination of factors triggering an imbalance in homeostasis throughout the central nervous system. However, most previous studies have explored the pathogenesis of depression from a single dimension without considering the combined effect of multidimensional factors. Secondly, the clinical manifestation varies greatly among individuals and patient groups are highly heterogeneous. MDD remains difficult to diagnose with accuracy in clinical practice and Mitchell *et al*. found that only about 50% of true cases had been diagnosed (Mitchell *et al.,* 2009). Objective indicators of MDD have not been identified and professionals rely primarily on phenomenological descriptions for diagnoses (McCarron *et al.*, 2021; Su and Si, 2022). Finally, the effectiveness of treatment requires improvement. The overall cumulative remission rate was only 67% in the STAR*D study (Rush *et al.,* 2006) and rates of recurrence remain high at 77.1% over the 6-year follow-up (Verduijn *et al*., 2017), partly because objective evaluations of long-term efficacy are lacking and treatments are often based on practical experience (McCarron *et al.,* 2021; Su and Si, 2022). Owing to MDD heterogeneity and various other features, no previous study has resolved these problems (Cai *et al.*, 2020; Pine, 2019) and a novel, nationally representative cohort study may be of assistance. Therefore, the Prospective Cohort Study of Depression in China (PROUD) has been designed with careful consideration of MDD features. The PROUD study will (1) collect multidimensional data; (2) undertake large-scale recruitment of patients with different features from 24 provinces and cities; (3) collect data on clinical manifestation using a standardized procedure and (4) observe and describe the course of MDD over long-term follow-up.

Prospective cohort studies focusing on depression have been conducted in other countries and we have learned from their strengths and limitations while designing the PROUD study. The Netherlands Study of Depression and Anxiety (NESDA) and the Texas Resilience against Depression (T-RAD) study are two such studies. NESDA is an ongoing longitudinal naturalistic cohort study, investigating the course and consequences of depressive and anxiety disorders. Its design integrated biological and psychosocial perspectives within an epidemiological framework (Penninx *et al*., 2021; Penninx *et al.,* 2008). T-RAD includes two 10-year natural history prospective studies and aims to uncover multimodal factors predictive of onset and progression of depressive disorder, treatment responsivity and to identify subgroups of depression and facilitate precise diagnosis and effective intervention (Trivedi *et al.,* 2020). Major strengths of both studies include long-term follow-up and the collection of comprehensive multidimensional data. However, both studies have the limitations of a comparatively small sample size (3,328 for the NESDA study reported in 2021 and 2,500 for each study of the T-RAD) and fixed follow-up schedules which make timely capture of unexpected critical events leading to mood changes in patients difficult (Penninx *et al.*, 2021; Trivedi *et al.*, 2020). The PROUD study is designed as a 3-year cohort study collecting multidimensional data, biospecimens, neuroimaging and electroencephalogram (EEG) data, but with a larger sample of 14,000 patients and more flexible follow-up schedule and strategy, including external visits prompted by qualifying events.

There have been previous cohort studies of depression in China but the PROUD study has a larger scale and improved shareability of cohort data. The PROUD study aims to establish a nationally representative, high-quality, standardized depression cohort and to construct a comprehensive database for facilitation of further analyses and studies. It is hoped to promote the understanding of homeostatic medicine for MDD and facilitate the development of precise diagnoses and treatments, thus filling gaps in current research.

## Methods and Analysis

### Study design and settings

PROUD is a patient-based, nationally representative multicenter prospective cohort study with baseline and 3-year follow-up assessments. It will be carried out from January 2022 to December 2026 in 52 qualified tertiary hospitals in China, covering 24 provinces and cities: Beijing, Shanghai, Sichuan, Hunan, Hebei, Tianjin, Shandong, Guangdong, Yunnan, Zhejiang, Anhui, Jiangxi, Henan, Shenzhen, Shanxi, Fujian, Hubei, Jiangsu, Liaoning, Heilongjiang, Chongqing, Shaanxi, the Xinjiang Uygur Autonomous Region and the Inner Mongolia Autonomous Region, which are belong to five economic regions in China (Ma, 2009). Multidimensional data, including sociodemographic characteristics, clinical information, biospecimen, neuroimaging, cognitive function, electrophysiology and digital phenotypes, will be collected using standardized operating procedures and uniform equipment to guarantee quality across centers. Ethical approval was obtained from Beijing Anding Hospital, Capital Medical University, Beijing, China. The protocol has been registered in the Chinese Clinical Trial Registry platform (number: ChiCTR2200059053).

### Participants and eligibility criteria

**Fig. 1:**
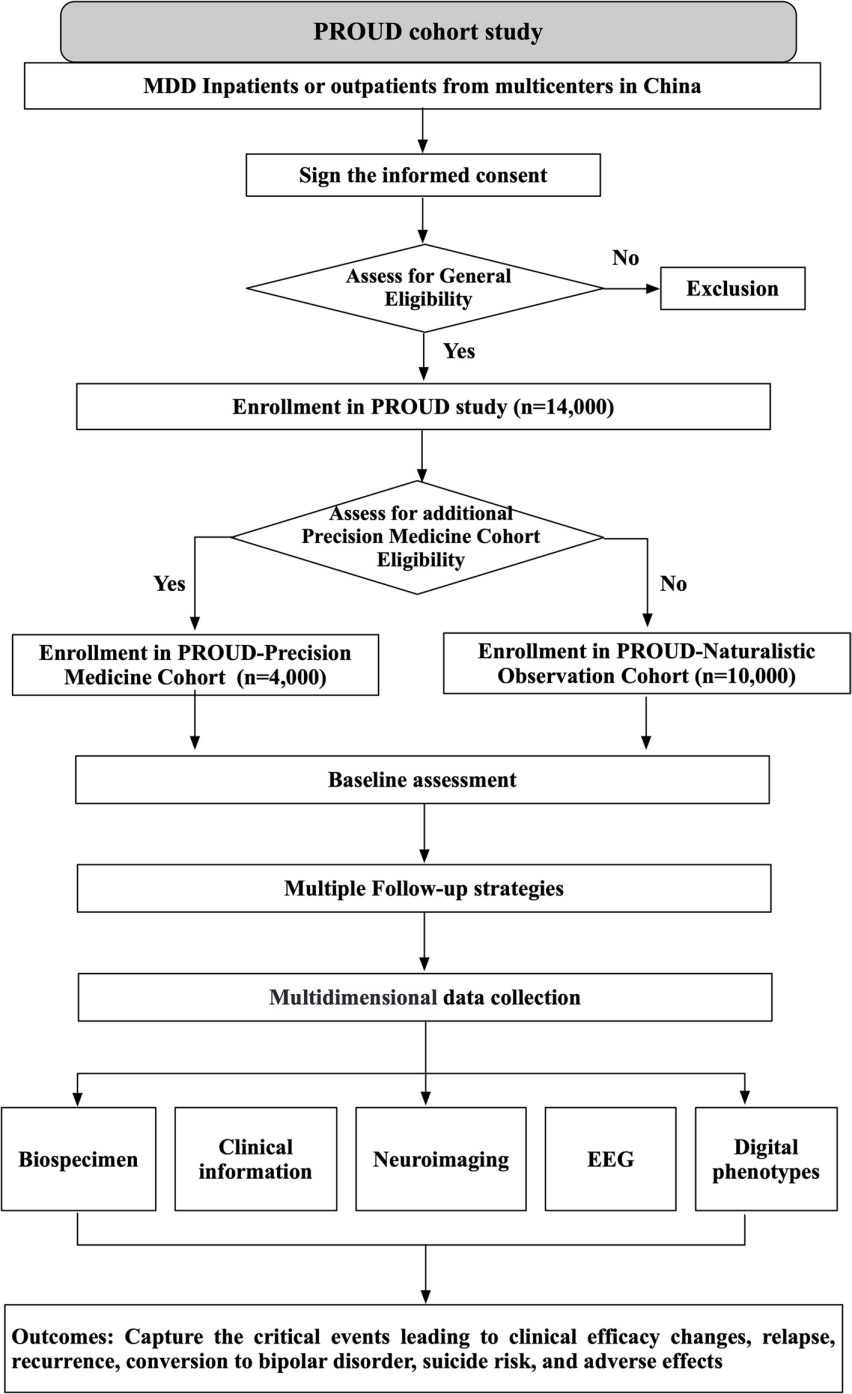
Flow chart of participant selection

The study population comprises MDD patients with diverse sociodemographic characteristics, disease stages and treatment regimens. About 14,000 eligible MDD patients will be recruited from 52 centers. MDD patients who have not received any treatment during the acute phase of the depressive episode will be included in the precision medicine cohort (PMC) of PROUD (*n*=4,000). Patients who meet the general eligibility criteria, but not the PMC criteria, will be included in the naturalistic observation cohort (NOC) of PROUD (*n*=10,000). There are no restrictions of the treatments and antidepressant agents for NOC cohort of PROUD. An additional 1,000 participants with no psychiatric history will be included as healthy controls (HC) and 1,000 participants with bipolar disorder will be included as diseased controls. The general flow chart of participant selection and evaluation can be found in Fig. 1.

### General inclusion criteria for PROUD

1. Males and females aged ≥16 years.
2. Inpatients or outpatients with a diagnosis of major depressive disorder based on the Diagnostic and Statistical Manual of Mental Disorders, 5th Edition (DSM-5).
3. Primary school education or above, able to understand the content of the scale.
4. Participation on a voluntary basis and provision of written informed consent.

### General exclusion criteria for PROUD

1. Comorbidities of clinically diagnosed schizophrenia, bipolar disorder, neurodevelopmental disorders, neurocognitive disorders or other major psychiatric diseases.
2. Severe medical conditions precluding participation in the study.
3. Any other conditions considered unsuitable for the study.

### Additional inclusion criteria for PROUD-PMC

1. Males and females aged 18-65 years.
2. A score of ≥14 on the 17-item Hamilton Depression Rating Scale (HAMD-17) at both screening and baseline.
3. Have not taken any antidepressant agents for at least 14 d before screening (fluoxetine therapy should be discontinued at least 28 d before screening).
4. Monotherapy for antidepressants planned.

### Additional exclusion criteria for PROUD-PMC

1. Depressive episodes secondary to a systemic disease or neurological disorder, such as depression due to hypothyroidism.
2. Received modified electric convulsive therapy (MECT), transcranial magnetic stimulation (TMS), deep brain stimulation (DBS) or vagus nerve stimulation (VNS) within 3 months before screening.
3. Withdrawal of psychotropic drugs did not equate to seven half-life periods before the screening.
4. Women with a positive blood human chorionic gonadotropin (HCG)/urine HCG test; men with reproductive potential and women of reproductive age unable to use contraception effectively and women who plan to become pregnant within 3 months of the start of the study.
5. Participated in any interventional clinical trial 3 months before screening.

### Data collection

An overview of data collection and assessments for field visits is shown in Table 1.

**Table 1:** Overview of assessments during field visits

Category	Components	Field visits									
		Baseline	2 w^a^	8 w^a^	12 w^a^	24 w^a^	52 w	76 w^a^	104 w	128 w^a^	156 w
**Sociodemographic, Lifestyle**											
Sociodemographic characteristics	Sex, age, ethnicity, weight, height, education, occupation	**×**									
lifestyle and diet habits	Defecation, smoking, drinking, dietary habits, water intake, use of antibiotics and probiotics	**×**	**×**	**×**	**×**	**×**	**×**	**×**	**×**	**×**	**×**
**Clinical**											
History of present illness	Date of the first onset, date of the current illness onset, total number of episodes, family histories	**×**									
Physical examination	Blood pressure, pulse, temperature, respiration and other abnormal results	**×**	**×**	**×**	**×**	**×**	**×**	**×**	**×**	**×**	**×**
Comorbidities	Diagnoses, onset time, remission time, treatments	**×**	**×**	**×**	**×**	**×**	**×**	**×**	**×**	**×**	**×**
Treatment	Physical therapy, psychotherapy, psychiatric medications	**×**	**×**	**×**	**×**	**×**	**×**	**×**	**×**	**×**	**×**
Adverse events	Adverse events, concomitant medications		**×**	**×**	**×**	**×**	**×**	**×**	**×**	**×**	**×**
**Symptoms evaluation**											
HAMD-17	Hamilton Depression Rating Scale for Depression -17-item (observer-rated)	**×**	**×**	**×**	**×**	**×**	**×**	**×**	**×**	**×**	**×**
HAMA	Hamilton Anxiety Scale (observer-rated)	**×**	**×**	**×**	**×**	**×**	**×**	**×**	**×**	**×**	**×**
BPRS-4	Brief Psychiatric Rating Scale, BPRS 4-items (observer-rated)	**×**	**×**	**×**	**×**	**×**	**×**	**×**	**×**	**×**	**×**
C-SSRS	Columbia-Suicide Severity Rating Scale (observer-rated)	**×**		**×**	**×**	**×**	**×**	**×**	**×**	**×**	**×**
QIDS-SR16	Quick Inventory of Depressive Symptomatology-Self-Report (self-rated)	**×**	**×**	**×**	**×**	**×**	**×**	**×**	**×**	**×**	**×**
SHAPS	Snaith-Hamilton Pleasure Scale (self-rated)	**×**	**×**	**×**	**×**	**×**	**×**	**×**	**×**	**×**	**×**
GAD-7	Generalized Anxiety Disorder (self-rated)	**×**	**×**	**×**	**×**	**×**	**×**	**×**	**×**	**×**	**×**
PDQ-D5	Perceived Deficit Questionnaire for Depression 5-item (self-rated)	**×**	**×**	**×**	**×**	**×**	**×**	**×**	**×**	**×**	**×**
HCL-33	Hypomania Check List (self-rated)	**×**	**×**	**×**	**×**	**×**	**×**	**×**	**×**	**×**	**×**
FISER/GRSEB	Frequency and Intensity of Side Effects Rating/Global Rating of Side Effects Burden (self-rated)		**×**	**×**	**×**	**×**	**×**	**×**	**×**	**×**	**×**
PRISE	Patient Rated Inventory of Side Effects (self-rated)		**×**	**×**	**×**	**×**	**×**	**×**	**×**	**×**	**×**
PAQ	Patient Adherence Questionnaire (self-rated)		**×**	**×**	**×**	**×**	**×**	**×**	**×**	**×**	**×**
SDS	Sheehan Disability Scale (self-rated)	**×**		**×**	**×**	**×**	**×**	**×**	**×**	**×**	**×**
MIDAS	Migmine Disability Assessment Questionnaire (self-rated)	**×**			**×**		**×**		**×**		**×**
HIT-6	Headache Impact Test-6 (self-rated)	**×**			**×**		**×**		**×**		**×**
MAES	Modified Apathy Estimate Scale (observer-rated)	**×**					**×**		**×**		**×**
CFS-11	Chalder Fatigue Scale-11 (self-rated)	**×**					**×**		**×**		**×**
PSS	Perceived Stress Scale (self-rated)	**×**					**×**		**×**		**×**
CTQ	Childhood Trauma Questionnaire (self-rated)	**×**									
EPQ	Eysenck Personality Questionnaire (self-rated)	**×**									
BPX	Bipolarity index (observer-rated)	**×**									
Cognitive evaluation	The Chinese Brief Cognitive Test (C-BCT)	**×**		**×**			**×**		**×**		**×**
**Biomarker**											
Biospecimen	whole blood, urine, stool, saliva	**×**	**×**	**×**			**×**		**×**		**×**
Neuroimaging	Resting-state fMRI, T1, DTI, Tasks	**×**		**×**			**×**		**×**		**×**
EEG	Resting state EEG, Tasks	**×**		**×**			**×**		**×**		**×**
Digital phenotyping	Portable EEG, heart rate, body temperature, sleep, voice, facial expression, eye movement, gait	**×**		**×**			**×**		**×**		**×**

^a^Additional visits for precision medicine cohort. *W* week

#### Sociodemographic information and lifestyle habits

Sociodemographic information will be collected through a structured self-report questionnaire at baseline, including birth date, gender, height, weight, marital status, education level, occupational status, household income, living area, left-behind children experience and experience of being an only child. Also, data on lifestyle and dietary habits such as defecation, smoking, drinking, dietary habits, water intake and the use of antibiotics and probiotics will be collected. Information will also be collected about the history of the present illness, physical examination results, comorbidities, treatments and adverse events.

#### Clinical assessment

Participants will be interviewed and diagnosed by a psychiatrist at baseline using the Mini-International Neuropsychiatric Interview (MINI). Diagnosis will be classified according to DSM-5 and MINI as depression with anxious distress, mixed features, melancholic features, atypical features, psychotic features, seasonal pattern or peripartum onset.

The following scales will be used to assess clinical symptoms and function at baseline: the HAMD-17 (Hamilton, 1960; Zheng *et al.*, 1988), Hamilton Anxiety Scale (HAMA) (Hamilton, 1959; Tang and Zhang, 1984), Brief Psychiatric Rating Scale (BPRS) (Faustman and Overall, 1999), Columbia-Suicide Severity Rating Scale (C-SSRS) (Posner *et al*., 2008), Bipolarity Index (BPX) (Ma *et al.*, 2016) and Modified Apathy Estimate Scale (MAES) (Starkstein *et al.,* 1992).

The self-report measures include the Quick Inventory of Depressive Symptomatology-Self-Report (QIDS-SR16) (Liu *et al*., 2013; Rush *et al*., 2003), Snaith-Hamilton Pleasure Scale (SHAPS) (Snaith *et al*., 1995), Generalized Anxiety Disorder-7(GAD-7) (Hidalgo and Sheehan, 2012), Perceived Deficit Questionnaire for Depression 5-item (PDQ-5) (Shi *et al.*, 2017; Sullivan *et al.*, 1990), Hypomania Check List (HCL-33) (Feng *et al.*, 2016; Wang *et al*., 2018), Frequency and Intensity of Side Effects Rating/Global Rating of Side Effects Burden (FISER/GRSEB) (Wisniewski *et al.,* 2006), Patient-Rated Inventory of Side Effects (PRISE) (Rush *et al.,* 2004), Patient Adherence Questionnaire (PAQ) (Mojtabai *et al*., 2012), Sheehan Disability Scale (SDS) (Sheehan, 1986), Childhood Trauma Questionnaire (CTQ) (Bernstein *et al.*, 2003), Eysenck Personality Questionnaire (EPQ) (Eysenck and Eysenck, 1975; Gong, 1984), Remote Follow-Up Checklist (RFC), Patient Health Questionnaire (PHQ-9) (Kroenke *et al.*, 2001; Wang *et al.,* 2014), Mood Disorder Questionnaire (MDQ) (Hirschfeld *et al.*, 2000), Ask Suicide-Screening Questions (ASQ) (Horowitz *et al.,* 2012), Life-chart Methodology (LCM) (Denicoff *et al.*, 2000), Migmine Disability Assessment Questionnaire (MIDAS) (Stewart *et al*., 2001), Headache Impact Test-6 (HIT-6) (Kosinski *et al.,* 2003), Chalder Fatigue Scale-11 (CFS-11) (Chalder *et al.,* 1993) and Perceived Stress Scale (PSS) (Cohen *et al.*, 1983).

#### Biospecimen collection

Biospecimens, including blood, urine, feces and saliva, will be collected to explore multi-omics interactions and reveal the pathophysiology of depression. Blood samples will be collected at baseline and scheduled visits with one pro-coagulation blood tube (6 mL), one PAXgene® Blood RNA Tube (2 mL) and two EDTA anticoagulation blood tubes (6 mL/tube). Urine samples will be collected with a urine cup (40 mL). A standardized kit (25mL) will be provided to collect fecal samples and maintain their integrity. Saliva samples will be collected with sterile, nuclease-free 5-mL conical screw-cap tubes. All blood, urine and fecal samples will be stored in −80°C freezers and transported through the cold chain to the PROUD biobank.

#### Cognitive assessment

The Chinese Brief Cognitive Test (C-BCT) will be used to assess four cognitive domains: speed of processing, attention, reasoning/problem-solving and working memory. The psychometric properties of the C-BCT are well-established, including large norms, test-retest reliability and consistency across cultures. Moreover, it has been validated against traditional paper-and-pencil tests that assess equivalent domains (Ye *et al.,* 2022).

#### Neuroimaging data

Neuroimaging will include T1- and T2-weighted structural imaging, fluid-attenuated inversion recovery (FLAIR), diffusion-weighted imaging (DWI), susceptibility-weighted imaging (SWI), resting-state functional MRI (fMRI) and diffusion tensor imaging (DTI). Anatomical and microstructural imaging will include a 3D high-resolution magnetization-prepared rapid gradient-echo (MPRAGE) sequence and a 64-direction diffusion tensor imaging sequence, respectively. Both resting-state and task-based fMRI data will be collected as part of the functional imaging protocol. The task-based BOLD fMRI signal will be used to analyze reward processing and emotional regulation.

Quantitative imaging, melanin imaging, arterial spin labelling and task-based fMRI will be optional in the PMC study. The task-based fMRI will include rumination, emotional recognition and social cognition tasks. All MRI scans will be performed using a 3.0T scanner (Siemens, GE and Philips) by trained investigators following a standardized protocol.

#### Electroencephalogram data

EEG data will be acquired using standard pre-specified hardware and software. The resting EEG will be recorded in two 5-min sessions while participants are relaxed with eyes open or closed. Event-related potentials (ERPs) will be elicited by activation tasks. The face-word Stroop paradigm was employed to assess cognitive and emotional regulation. The experiment requires a quiet environment with a comfortable temperature and humidity level and electromagnetic shielding. The EEG data will be recorded using the Neuroscan and Brain Products with 64 electrodes.

#### Digital phenotype data

Digital phenotype data, including portable EEG, heart rate, body temperature, sleep and other parameters, will be collected using wearable devices during daily life. Eye movement, gait, voice and facial expressions will be collected in the hospital during the visits, as the collection of these data requires space and specialized equipment.

### Follow-up strategies

#### General follow-up strategies for PROUD

All participants of the PROUD cohort study will be followed up by trained investigators at least once a year for at least 3 years, through a combination of field visits, remote visits, telephone visits and patient self-reporting.

1. Field visits: All patients will be required to make field visits at baseline and at the end of years 1, 2 and 3 (Table 1). Demographic, clinical, biological, neuroimaging, EEG and digital phenotype data will be collected. If a patient is unable to make a field visit at the scheduled time due to extenuating circumstances, a telephone visit will be performed by an investigator.
2. Remote visits: A remote follow-up visit will be conducted by sending a RFC to the patient via a mobile device every 4 weeks to capture patient mood swings. The checklist includes seven questions on depression, anxiety, suicide/self-injury and mania/hypomania.
3. Telephone visits: Telephone visits will be conducted every 3 months to gather information on mood conditions, treatments, medication use and outcomes to ensure patient compliance and to acquire timely information regarding their condition.
4. Patient self-reports: Patients will actively report their conditions, such as mood swings, somatic diseases, treatment information and adverse events, via WeChat, SMS, telephone calls and in-person visits to the hospital.

#### Additional follow-up strategies for PROUD-PMC

**Fig. 2:**
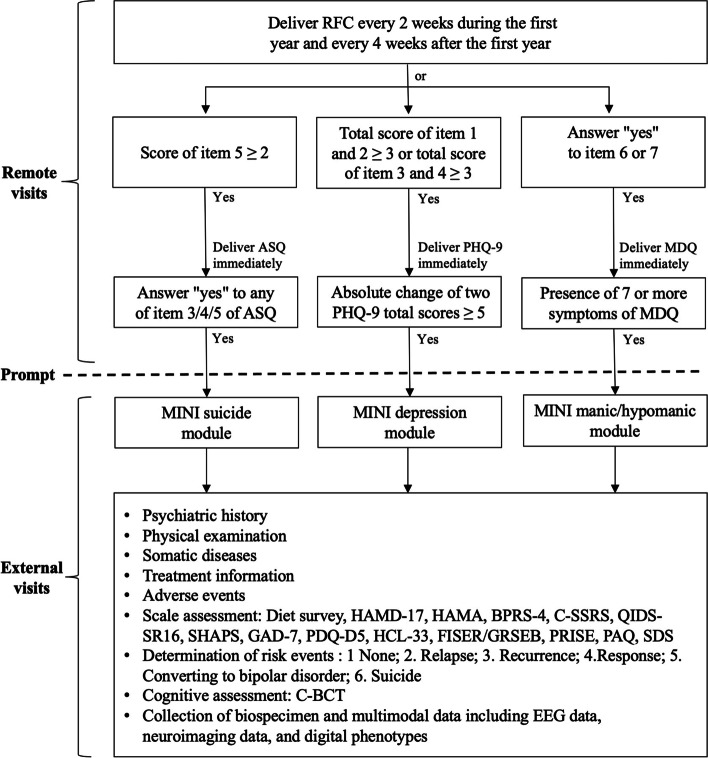
Logic diagram of external visits

PROUD-PMC is based on PROUD's follow-up strategies but with extra field visits at the end of weeks 2, 8 and 12. The frequency of remote visits is increased to once every 2 weeks throughout the first year. PROUD-PMC includes external visits to capture the critical points in depression progression and chart the disease trajectory. After 12 weeks, all PROUD-PMC cohort study participants will be followed up by sending regular RFC reminders via a mobile device (see the section on remote visits). An external visit will be scheduled if the RFC scores reach certain thresholds. The logic diagram of external visits can be found in Figure 2.

1. If the combined score of RFC items 1 and 2 or of items 3 and 4 is ≥ 3, the PHQ-9 will immediately be delivered to the mobile device. An external visit will be scheduled if the difference between the total PHQ-9 score at that time and the previous total score is ≥ 5, indicating possible depression relapse or recurrence. A decrease in the PHQ-9 score of ≥ 5 points may indicate amelioration of the patient’s depressive condition.
2. If RFC item 5 is scored ≥ 2, the ASQ will be delivered immediately and an answer of “yes” for any of items 3–5 indicates high risk of suicide and an external visit will be requested.
3. If the answer to RFC item 6 or 7 is "yes", the MDQ will be delivered immediately. If ≥ 7of the MDQ's 13 screening symptoms are present, indicating risk of mania/hypomania, an external visit will be scheduled.

The external visit will be in the form of a field visit which consists of the following: (1) Re-evaluation of the relevant module of the MINI; (2) Collection of information on psychiatric history, physical examination results, somatic diseases, treatment and adverse events; (3) Scale assessment; (4) Re-evaluation of the event that prompted the external visit; (5) Cognitive assessment and (6) Collection of biospecimen and multimodal data, including EEG, neuroimaging and digital phenotype data. These data will only be collected once if a patient has two or more risk events of the same type throughout the follow-up period.

### Outcomes

#### Primary outcomes

Primary outcomes are depressive symptom changes, as measured by the HAMD-17 total scores in the acute, maintenance and long-term follow-up phases compared with baseline. Clinical efficacy in different phases is defined as follows:

1. Early onset rate: a reduction of HAMD-17 total score ≥20% from baseline by the end of week 2.
2. Response rate: a reduction of HAMD-17 score ≥50% from baseline. Additionally, a stable response is defined as a reduction of two consecutive HAMD-17 total scores ≥50% from baseline at weeks 8 and 12.
3. Remission rate: a HAMD-17 score ≤7 in the acute phase. Stable remission is defined as two consecutive HAMD-17 scores ≤7 at weeks 8 and 12.
4. Relapse: a HAMD-17 score ≤7 or a reduction of HAMD-17 score ≥50% at the end of the acute treatment, lasting for <2 months, followed by a HAMD-17 score ≥14 in combination with meeting the diagnostic criteria for MDD of DSM-5.
5. Recurrence: a HAMD-17 score ≤7 at the end of the acute treatment, lasting for >2 months, followed by a HAMD-17 score ≥14 in combination with meeting the diagnostic criteria for MDD of DSM-5.

#### Secondary outcomes

1. Hospitalization for psychiatric episodes or emotional events.
2. Conversion to bipolar disorder: DSM-5-diagnosed manic or hypomanic episodes.
3. High risk of suicide: score on the third item of the HAMD-17 ≥3 or answering “Yes” to item 4 or 5 of the C-SSRS.
4. Adverse effects of drug treatment and cumulative drug exposure.
5. Adherence to treatment, as measured with the PAQ. Not taking prescribed drugs ≥70% of the time is considered to indicate non-adherence.
6. Changes in social functions in the acute, consolidation and maintenance phases compared with those at enrollment.
7. Changes in cognitive function in the acute, consolidation and maintenances phase compared with those at enrollment.
8. Changes in multi-omics, neuroimaging, electrophysiology and digital phenotype indicators.

### Data management

**Fig 3:**
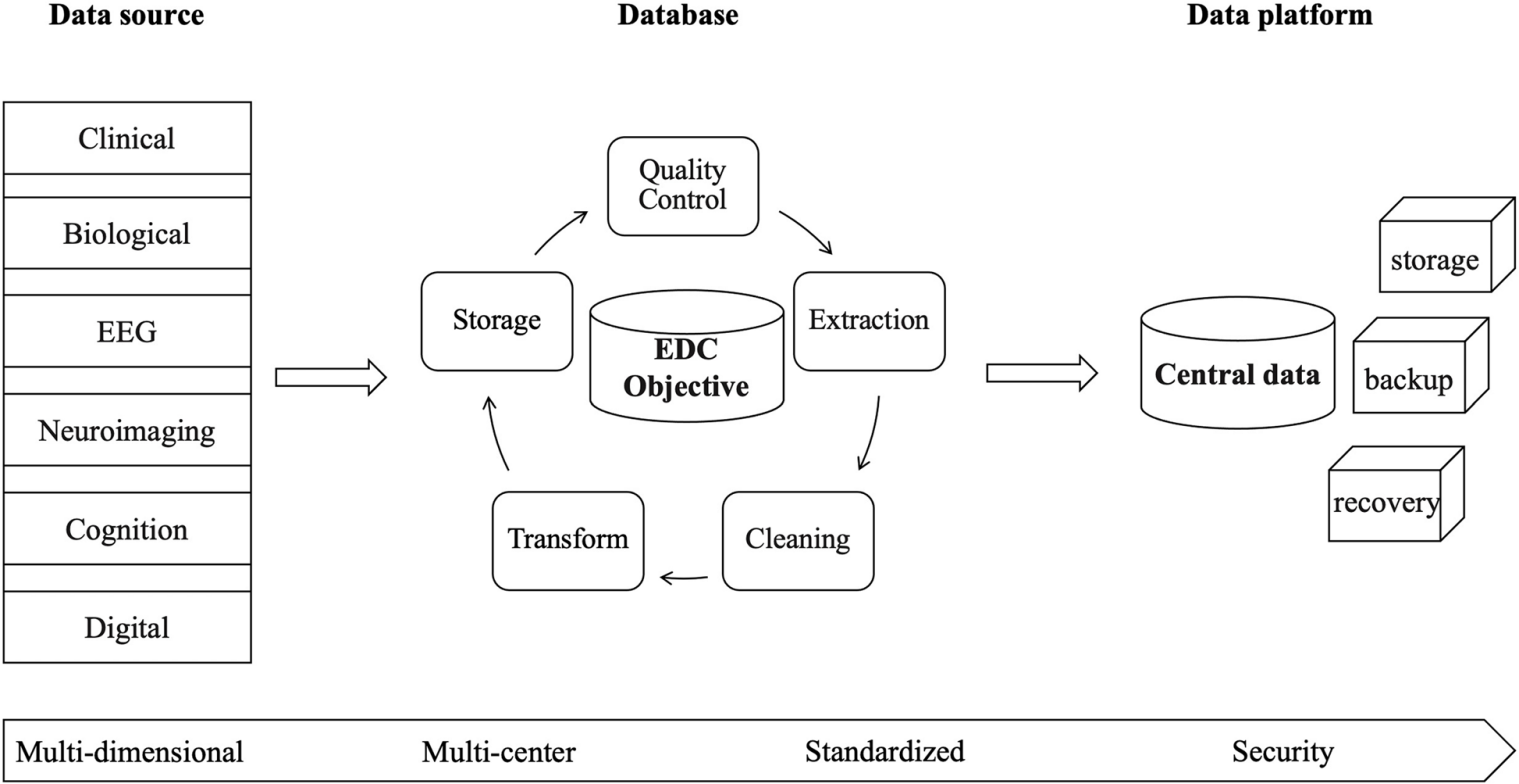
Data management

A multi-source central data platform will be established to accommodate the multidimensional data included in the study (Figure 3). Clinical and objective databases will be used. The clinical database contains demographic, clinical and cognitive data collected by the electronic data capture (EDC) system, while the objective database contains biospecimen, neuroimaging, EEG and digital phenotype data. The central data platform will extract multidimensional data from each data management system (e.g. neuroimaging system, EEG system and biobank). We have established standardized disease-specific cohort study dataset frames and data elements based on the Clinical Data Interchange Standards Consortium (CDISC), Standard Data Tabulation Model (SDTM) and Chinese national or industry terminology and specifications. All centers will collect data following standard operating procedures (SOP). Then, the data collected at each center will be uploaded to the local platform, transformed into a uniform format and transferred to the central data platform. Quality control will be applied at every step in this process. Each participant will be assigned a unique identification number. Other functions of the central data management platform include backup and recovery, ensuring data security and confidentiality and quality control checks on all data.

## Data analysis

Comprehensive data, amounting to thousands of variables across several modalities, will be collected in this project. Thus, we will be able to perform exploratory, data-driven analyses, and targeted, hypothesis-driven researches. These analyses and researches will help us investigate the trajectory of depression, identify clinical and biological subtypes of MDD, and construct prediction models of primary and secondary outcomes.

Several methods will be available to investigate changes in symptoms and facilitate an understanding of the disease trajectory, such as repeated-measures analysis of variance/mixed-effects model, time series analysis, latent variable growth curve model, multi-layer linear model and dynamic Bayesian network model. Cluster analysis, principal component analysis and other algorithms may be used to identify clinical and biological subtypes of depression.

Methods considered for the features' selection before the predictive model construction include the least absolute shrinkage and selection operator (LASSO), hierarchical LASSO and elastic net. Machine learning algorithms will then be used to build a model integrating multimodal biomarkers (multi-omics, EEG, and neuroimaging markers) and clinical features. Receiver operating characteristic (ROC) curve, cross-validity, net reclassification improvement (NRI) and integrated discrimination improvement (IDI) will be calculated to estimate and improve the performance of the model.

## Discussion

MDD is responsible for the highest burden of disease of any non-fatal disease worldwide, but its etiological mechanisms remain unclear and we are unable make the correct diagnosis and provide effective treatment with consistency. PROUD aims to accelerate homeostatic and precision medicine by establishing a prospective cohort study. This study has two significant strengths. (1) It will recruit a large, multidimensional cohort representative of the Chinese population: a total of 14,000 patients will be included, recruited from 52 tertiary hospitals across 24 provinces and cities in China. Furthermore, multimodal data will be collected, including clinical phenotypes, multi-omics, neuroimaging, neurophysiology and digital phenotypes. (2) This study will track mood changes and investigate the progression of MDD. External visits will be scheduled automatically whenever certain criteria are met and will be able to capture the patients' mood swings.

The present study will develop standard operating and quality control procedures for the collection of multidimensional data, and construct a data platform to ensure standardization and shareability of data. Data from multidimensional sources will be integrated into a central data platform, thus aiding the management and future application of the data.

It is clear that this study may also encounter certain difficulties. In response to the Corona Virus Disease 2019 (COVID-19), we have made the “Emergency Plan for Prevention and Control of Novel Coronavirus Pneumonia Outbreak”. If an outbreak strikes, we will actively coordinate resources, prioritize the security of the patients and researchers and try to minimize the impacts on progress while maintaining the quality of the study.

In conclusion, the PROUD study will construct and provide a comprehensive database that will facilitate further analyses and studies and the development of homeostatic and precision medicine in China.

## Data Availability

The data generated during the study are not publicly available due to data security and privacy but are available from the corresponding author on reasonable request.
